# Public knowledge, attitudes, and perceptions toward genetically modified foods in Lebanon

**DOI:** 10.1080/21645698.2025.2450852

**Published:** 2025-01-15

**Authors:** Hussein F. Hassan, Hasan Yassine, Ahlam Chaaban, Ahmad Chehaitly, Zeinab Skaineh, Nagham Cherri, Sahar Moussawi, Nour Baytamouni, Philippe Hussein Kobeissy, Hani Dimassi, José-Noel Ibrahim

**Affiliations:** a Department of Nutrition and Food Science, School of Arts and Sciences, Lebanese American University, Beirut, Lebanon; b Department of Biological Sciences, School of Arts and Sciences, Lebanese American University, Beirut, Lebanon; c U1179 INSERM, END-ICAP, UFR des Sciences de la Sante-Simone Veil, Universite de Versailles Saint-Quentin-en-Yvelines, Montigny-le-bretonneux, France; d School of Pharmacy, Lebanese American University, Byblos, Lebanon

**Keywords:** Acceptance, attitude, education, food safety, GMO, knowledge, perception

## Abstract

Genetically modified foods (GMFs) have garnered significant attention due to their implications for health, agriculture, and food security. Understanding knowledge and perceptions of GMFs is essential, as these factors influence acceptance and attitudes. This study, the first of its kind in Lebanon, aimed to assess the knowledge, attitudes, and perceptions regarding GMFs and to explore their association with socio-demographic characteristics. A cross-sectional study was conducted with 1,001 participants who completed a 50-item questionnaire, followed by a 15-minute educational session on GMFs. Statistical analysis was performed using SPSS. Prior to the educational session, participants had an average knowledge score of 60.3 ± 17.4%, which significantly increased to 83.0 ± 15.8% afterward. Attitude and perception scores improved from an average of 30.3 ± 25.1% pre-intervention to 38.9 ± 12.4% post-intervention. Females demonstrated significantly higher knowledge scores, and educational attainment was positively correlated with knowledge levels both pre- and post-intervention. Younger participants and those in health-related fields scored higher before the intervention, while unemployed individuals scored lower. Additionally, higher educational levels and health-related educational backgrounds were linked to better attitudes and perceptions pre-intervention, while unemployment correlated with lower scores. Interestingly, multivariate regression analysis indicated that being under 35 years of age, having a health-related educational background, and holding a university degree were predictors of higher GMF knowledge. Consequently, individuals with initially lower knowledge level benefited the most from the educational intervention, exhibiting the greatest knowledge increases post-education. Our findings underscore the importance of targeted educational initiatives to bridge knowledge gaps and address misconceptions regarding GMFs.

## 1. Introduction

Genetic modification refers to biological techniques associated with the change of the genome and genetic machinery of living organisms.^1^ According to the World Health Organization (WHO), genetically modified organisms (GMOs) are plants, animals, or microorganisms whose genetic material (DNA) has been modified in a manner that does not happen naturally through reproduction or natural genetic exchange.^2^ Therefore, “GM foods” (GMFs) refer to foods produced from GM plants or animals, such as golden rice, arctic apples, and *Bacillus thuringiensis* corn.^3^ In the last three decades, the usage of GMOs has increased by more than a hundredfold.^4,5^ Indeed, GMFs offer many advantages over organic crops, including resistance to pathogens, drought tolerance, and increased economic benefits.^6^ Nevertheless, an international concern pertinent to the risks associated with GMOs to both environmental and human health has been raised.^7^ As such, a need for regulating the usage and spread of GMOs was depicted by the Cartagena Protocol on Biosafety, which took effect in 2003.^8^

Different governments and unions have responded to the perceived risks of GMOs in various ways, primarily through diverse legislation measures. For instance, the EU is considered to have one of the strictest GMO regulations based on the assumption that GMFs are more dangerous than their organic counterpart.^9^ However, the attitude of Indonesian scientists toward GMOs was more positive due to the familiarity with the technology.^10^ Uganda consumers, similar to Indonesia, tend to have a more positive attitude toward GM bananas, valuing their added nutritional value, affordability, and quality over its presumed potential health risks.^11^ In Kenya, a cross-sectional survey of 216 participants showed that over half believe GMFs are safe, have low threats to health, and are a solution to food insecurity in Africa.^3^ Interestingly, in China, findings concerning GMO commercialization are controversial and somehow contradictory. For instance, a study conducted among urban consumers in developed areas of China showed minimal resistance to GMFs due to the majority of them being familiar with GMOs.^12^ On the contrary, a nationwide study exploring the public perception of GMFs revealed that most consumers were either neutral or negative toward GMFs, mainly because of being unfamiliar with GM technology or receiving negative information from the internet or media.^13^ Lastly, the MENAT (Middle East, North Africa, and Turkey) region tends to exhibit negative attitudes toward GMFs due to the poor knowledge of the technology.^14^ Therefore, it seems that consumer attitudes toward GMFs are complex and intertwine with the knowledge of the technology, perceptions of risks and benefits, and individual lifestyle and moral beliefs.

Although Lebanon is not a producer of GMOs, the country has addressed the issue by developing a biosafety framework.^15^ Interestingly, the country has a national legislation concerning GMO imports under Article 6 of the “Plant Quarantine and Phytosanitary Measures” Law # 778 dated 28/11/2006.^16^ Previous research has indicated that GMFs, particularly soybeans, have been present in the Lebanese market.^15^ Nonetheless, no studies have explored the Lebanese individuals’ knowledge of GMFs and its impact on their attitudes and perceptions of the risks, benefits, purchase intentions, and consumption of GMFs.

Due to the rising global food security challenges and the ongoing decade-long debates on GMFs, the present study aims to investigate the knowledge, attitudes, and perceptions of the general population in Lebanon toward GMFs. Moreover, it intends to explore the effects of education and advertisement on the public knowledge and, consequently, its influence on their attitudes and acceptance levels of GMFs. The findings will reflect the public’s stance, behavior, and response as to whether GMF production and consumption should be legalized in Lebanon.

## 2. Materials and Methods

### Study Design and Population

This study was conducted according to the Declaration of Helsinki and approved by the Institutional Review Board of the Lebanese American University (IRB #: LAU.SAS.JI1.17/Jan/2024). The sample consisted of Lebanese individuals aged between 18 and 65 years old. The sample size was estimated according to the Institutional Effectiveness and Assessment Board, which recommends a sample of 1,000 to ensure the representativeness of the sample in large populations.^17^

**Figure 1.**
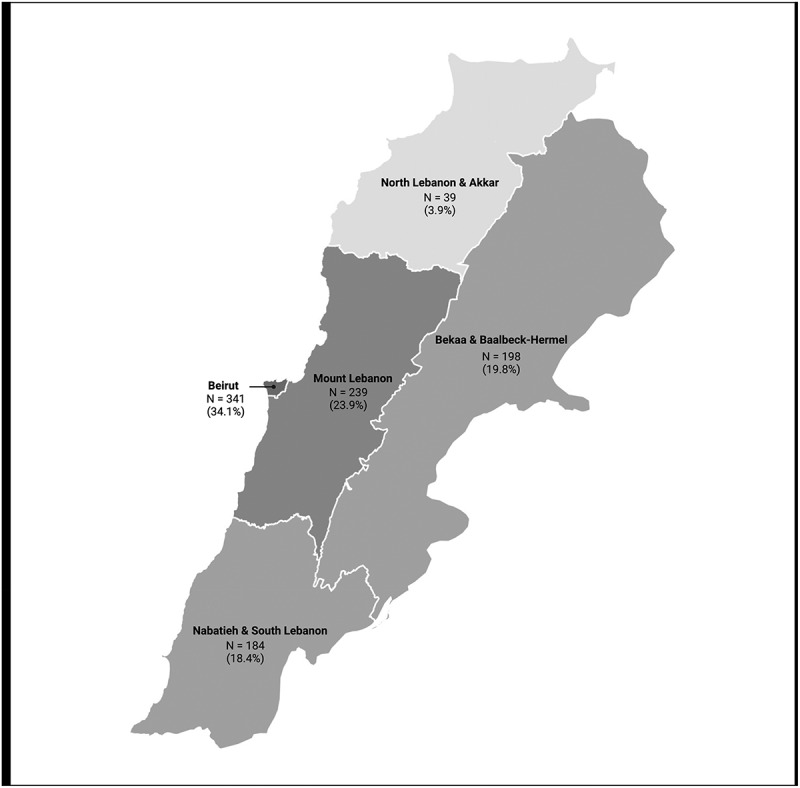
Map of the study area showing the distribution of the participants across the different governorates.

A total of 1,500 individuals from across Lebanon were conveniently approached to participate in the study, ensuring a proportional distribution across the governorates based on population size in each (Figure 1). Approximately 10% (*N* = 151) did not accept to participate in the study, mainly due to lack of motivation, interest in the research topic, or time availability.

Face-to-face meetings were arranged with individuals interested in participating based on their availability. After explaining the nature and purpose of the research, respondents were asked to provide their written consent to participate voluntarily in the research project. However, 349 individuals lost interest mid-survey and failed to complete the questionnaire, hence making the final sample size of 1,001 participants.

### Questionnaire

Data were collected between January and August of 2024 using a 50-item structured questionnaire designed based on previous studies and adapted for the Lebanese context.^18,19^ The questionnaire was thoroughly reviewed and approved by multiple experts who have considerable expertise in the field before being translated into Arabic and then back-translated by bilingual experts to ensure accuracy. Both language versions were pilot-tested to gather feedback on the clarity of instructions, comprehension of questions, and difficulty in answering them. The questionnaire was then completed in either Arabic or English, depending on the participants’ preference. The first part of the questionnaire focused on the sociodemographic characteristics, including age, sex, education, residence, and occupation. The following sections explored the participants’ subjective and objective knowledge of GMFs, perceived risks and benefits, attitudes, and willingness to buy and consume GMFs. Subjective knowledge was assessed using a 5-point Likert scale [1=Not knowledgeable at all, 2=Somehow unknowledgeable, 3= Neutral, 4=Somehow knowledgeable, 5=Extremely knowledgeable]. Participants were then asked seven “true” and “false” questions to assess their objective knowledge on GMFs. The correct answer was scored “1” and the incorrect answer was given a “0.” Perceived risks, benefits, attitudes, and acceptance of GMFs were assessed using a 5-point Likert scale [1=Strongly disagree, 2=Disagree, 3=Neutral, 4=Agree, 5=Strongly agree]. Correct answers were summed up and both knowledge and attitudes/perceptions scores were converted over 100%.

### Educational Session

At the end of the questionnaire, participants received a 15-minute educational session on GMFs, narrated by trained interviewers under the guidance of the principal investigator. The purpose aimed to improve participants’ objective knowledge and to assess how their increased knowledge would impact their perceptions and attitudes toward the use and consumption of GMFs. The content of the educational session was developed using different scientific resources.^20,21^ It included definitions and examples of GMFs, as well as their benefits on health and the environment, and potential associated risks.^18,19^ After completing the session, the participants were asked how informative the educational session was using a 5-point Likert scale [1=Not informative at all; 2=Not that informative; 3=Neutral; 4=Somehow informative; 5=Extremely informative]. In addition, their objective knowledge, perceived risks and benefits, and attitudes were assessed, and scores were computed and compared to those obtained before the educational session.

### Data Confidentiality

Information obtained from the survey was de-identified and kept anonymous. To preserve privacy, information collected from the questionnaire was linked to a secret code. Participants’ names were only recorded on the informed consent form. The informed consent and hard copy records were carefully protected in a locked file cabinet, with access limited to the principal investigator and authorized personnel. Electronic copies were saved in an Excel sheet on the laptop of the principal investigator secured with a password.

### Statistical Analysis

Data collected were coded and entered into SPSS software, version 29, for analysis. Knowledge scores were computed by summing responses to knowledge-based questions both before and after the educational intervention. Perception scores were calculated similarly. To assess differences in knowledge and perception scores by participants’ characteristics, independent samples t-tests and one-way ANOVA were employed, with assumptions of normality verified prior to analysis. Pearson’s correlation coefficient (r) was used to measure the strength and direction of relationships between numerical scores. Two linear regression models were constructed to evaluate the independent effects of participants’ characteristics on knowledge scores at baseline and on changes in knowledge scores post-intervention. Statistical significance was set at *p* < 0.05.

## 3. Results and Discussion

### Demographic Characteristics of the Study Population

A total of 1,001 valid questionnaires were included in the study. Of the respondents, 63.6% were females, 34.1% from the capital Beirut and its suburbs, 70.5% holding a university degree, 66.7% below 35 years old, 20.5% having health-related degree, and 65.0% single (Table 1).

**Table 1.** Demographic characteristics of the study population (*N* = 1,001).

Demographic Characteristics	N	%
**Gender**		
Male	364	36.4
Female	637	63.6
**Residential Governorate**		
Beirut & suburbs	341	34.1
Mount Lebanon	239	23.9
Bekaa/Baalbeck-Hermel	198	19.8
South Lebanon/Nabatieh	184	18.4
North Lebanon/Akkar	39	3.9
**Educational Level**		
Below elementary	24	2.4
Elementary/Primary school or equivalent	43	4.3
High school diploma or equivalent	228	22.8
Bachelor’s degree	564	56.3
Master’s degree	105	10.5
Doctorate degree	37	3.7
**Monthly household income ($)**		
<50	29	2.9
50–200	109	10.9
200–999	343	34.3
1000–2999	239	23.9
≥3000	166	16.6
Missing	115	11.5
**Age**		
18–24	560	55.9
25–34	108	10.8
35–44	95	9.5
45–54	116	11.6
55–64	76	7.6
65 and above	46	4.6
**Major of study**		
Health-related	205	20.5
Non-health related	330	33.0
Not applicable (due to educational level)	270	27.0
Missing/Not specified	196	19.6
**Area of residence**		
City	679	67.8
Town	195	19.5
Village	127	12.7
**Family status**		
Single	651	65.0
Having kids	310	31.0
Not having kids	38	3.8
Missing	2	0.2
**Working status**		
Unemployed/Retired	194	19.4
Student	504	50.3
Employed/Self-employed	301	30.1
Missing	2	0.2
**Field of work**		
Health-related	31	3.1
Non-health related	199	19.9
Not applicable (not employed)	191	19.1
Not applicable (student)	504	50.3
Missing/Not specified	78	7.8

### Knowledge of GMFs

Seven questions were asked to assess participants’ knowledge of GMFs, with pre- and post-educational responses presented in Table 2. Most participants in our study (91%) were aware of GM technology, aligning with findings by Catherine et al.,^3^ who reported 100% awareness in Kenya. Additionally, 62.0% and 60.0% of our participants knew that GMFs are not produced but are present in Lebanon, respectively. In contrast, 91.7% of participants in Kenya indicated that GMFs are available in the market, while in Saudi Arabia, majority of the respondents (73.4%) were not aware of GMFs in the market.^22^ Furthermore, 25.0% of participants in Kenya considered GM technology harmful, whereas in our study, 41.4% believed GMFs contain hazardous chemicals, and 69.1% thought that consuming GMFs could modify human genes. Finally, only 31% of our sample knew that there is a law that regulates GMF production and consumption, which is in agreement with data reported in Saudi Arabia (31%).^22^ Interestingly, the percentage of correct answers increased across all seven questions following the educational component.

**Table 2.** GMF knowledge questions/answers before and after the educational session.

GMF Knowledge Question (*correct option is shown in bold*)	Before		After	
	N	%	N	%
*GM foods exist*				
**TRUE**	911	91.0	991	99.0
FALSE	90	9.0	10	1.0
*GM foods contain hazardous chemicals*				
TRUE	587	58.6	180	18.0
**FALSE**	414	41.4	821	82.0
*Eating GM foods will modify a person’s genes*				
TRUE	309	30.9	122	12.2
**FALSE**	692	69.1	879	87.8
*It is possible to transfer genes from microorganisms to plants*				
**TRUE**	637	63.6	845	84.4
FALSE	364	36.4	156	15.6
*Currently, GM foods are produced in Lebanon*				
TRUE	380	38.0	164	16.4
**FALSE**	621	62.0	837	83.6
*GM foods are available in the Lebanese market*				
**TRUE**	601	60.0	641	64.0
FALSE	400	40.0	360	36.0
*In Lebanon, there is a law that regulates GM food production and consumption*				
**TRUE**	310	31.0	844	84.3
FALSE	691	69.0	157	15.7

The mean knowledge scores improved significantly (*p* < 0.001), rising from 60.3 ± 17.4% before to 83.0 ± 15.8% after the educational component. Females scored significantly higher than males (*p* < 0.05), and a higher educational level was associated with a significant increase in mean knowledge scores both before (*p* < 0.001) and after (*p* = 0.017) the educational component. Younger participants and those studying health majors scored higher before the intervention (*p* < 0.001), while unemployed individuals and those working in non-health related fields scored lower (*p* < 0.001 and *p* = 0.017 respectively) (Table 3).

**Table 3.** Relations between demographic characteristics and mean GMF knowledge score before and after the educational session.

Demographic Characteristics	Before		After	
	Mean	SD	Mean	SD
**Gender**				
Male	58.6	16.7	81.6	17.0
Female	61.3	17.6	83.6	15.0
*Sig.*	0.02		0.04	
**Residential Governorate**				
Beirut & suburbs	60.6	16.5	82.0	16.6
Mount Lebanon	63.3	18.2	82.8	15.5
Bekaa/Baalbeck-Hermel	57.6	17.6	84.2	14.3
South Lebanon/Nabatieh	59.0	17.4	84.3	16.2
North Lebanon/Akkar	59.7	16.7	81.7	15.4
*Sig.*	0.01		0.39	
**Educational Level**				
Uneducated	45.8	13.3	73.8	13.8
Elementary/Primary school or equivalent	54.5	16.3	81.7	16.6
High school diploma or equivalent	56.5	17.5	81.6	15.1
Bachelor’s degree	61.8	16.6	83.7	16.0
Master’s degree	63.9	18.2	85.4	15.1
Doctorate degree	67.2	19.0	83.0	17.1
*Sig.*	<0.001		0.017	
**Age**				
18–24	62.3	16.5	82.7	16.5
25–34	63.4	17.9	83.5	15.0
35–44	58.8	18.7	86.3	14.7
45–54	58.0	18.6	84.5	12.6
55–64	51.9	16.8	80.8	16.6
65 and above	51.2	14.3	79.5	16.1
*Sig.*	<0.001		0.08	
**Major of study**				
Health-related	67.4	15.4	86.4	14.8
Non-health related	60.5	16.7	84.4	15.2
*Sig.*	<0.001		0.136	
**Area of residence**				
City	60.2	17.2	82.2	15.8
Town	63.1	16.7	86.2	14.9
Village	56.5	18.5	82.7	16.6
*Sig.*	0.004		0.008	
**Working status**				
Unemployed/Retired	54.9	16.6	82.1	14.6
Student	62.4	16.7	82.8	16.2
Employed/Self-employed	60.2	18.2	84.1	15.8
*Sig.*	<0.001		0.356	
**Field of work**				
Health-related	68.2	18.0	88.5	14.0
Non-health related	59.9	18.0	84.4	15.7
*Sig.*	0.017		0.177	

The significant effect of education on the GMF knowledge confirms that education can effectively enhance understanding of complex topics like GMF. Interestingly, females scored significantly higher than males. This can be attributed to the fact that women often show a strong interest in health topics due in part to the broader representation of women in health-related and life sciences fields, where GMO topics are frequently covered. Additionally, since women traditionally play a significant role in food purchasing and meal preparation within households, they may encounter GMO information on food labels or through media focused on food safety. This consumer role likely increases their awareness and understanding of GMOs. Moreover, women are sometimes more engaged in public health campaigns and community discussions addressing health issues, which may further enhance their knowledge in this area. Research also suggests that women generally exhibit greater concern regarding food safety risks, leading them to seek information on GMOs to assess potential health and environmental impacts.^23^

On the other hand, unemployed participants and those in non-health-related fields scored significantly lower on GMF knowledge for a few key reasons. First, individuals in non-health-related fields may have less exposure to scientific discussions about GMFs, as their academic and professional backgrounds might not cover biotechnology, food safety, or public health topics where GMOs are frequently addressed. Second, unemployed participants may have fewer opportunities to engage with educational materials or professional networks where new information on GMOs is shared. Employment in health or science-related fields often provides access to industry updates, seminars, and other learning opportunities that keep professionals informed on topics like GMOs. Furthermore, without a professional or academic incentive to stay informed about health and food safety topics, these participants may have limited motivation to seek out information on GMOs independently. This was reported previously in food science studies in Lebanon assessing knowledge and attitudes.^24–33^

Notably, younger participants and those studying health-related fields scored significantly higher in the pre-educational phase (Table 3), potentially indicating preexisting exposure or interest in GMF within health-related curricula. This goes in line with the results of the multiple regression on the mean knowledge pre-educational intervention score, which showed that younger age (below 35), health-related educational background, and graduate studies are predictors of better GMF knowledge (Table 4). However, the absence of significant differences among these groups post-education suggests that the educational component successfully bridged initial knowledge disparities, bringing participants to a more uniform level of understanding. This was further supported by the results of the multiple regression analysis (Table 5) showing that the mean knowledge score increased 6–8 times among participants aged above 35, 5–8 times among those without graduate studies, and 4 times among non-health background, following the educational intervention.

**Table 4.** Predictors of knowledge on GMF.

	B	Std. Error	p-value
(Constant)	50.67	2.50	<0.001
Gender (*ref. Male*)			
Female vs Male	1.07	1.12	0.339
Governorate (*ref. Mount Lebanon*)			
Beirut	−3.01	1.40	0.032
Bekaa/Baalbeck hermel	−4.40	1.61	0.007
South Lebanon Nabatieh	−3.49	1.64	0.033
North Akkar	−3.00	2.88	0.297
Age (*ref. 55+*)			
Age = 18–24	8.57	1.75	<.0001
Age = 25–34	8.20	2.31	<0.001
Age = 35–44	4.21	2.34	0.072
Age = 45–54	4.09	2.19	0.061
Education (*ref. Non university*)			
Education=Bachelor’s degree	2.92	1.29	0.024
Education=Master’s degree	7.29	2.05	<0.001
Education=MD, PhD	8.49	3.07	0.006
Major (*ref. Non-health related*)			
Health-related	6.27	1.40	<0.001

**Table 5.** Multivariate analysis of change in knowledge on GMF.

	B	Std. Error	p-value
(Constant)	11.04	2.29	<0.001
Age (*ref. 34 and below*)			
35–44	8.06	2.24	<0.001
45–54	6.70	2.08	0.001
55–64	8.05	2.52	0.001
65 and above	7.03	3.32	0.034
Education (*ref. Graduate level*)			
Below elementary	6.19	4.67	0.185
Elementary/Primary or equivalent	7.00	3.67	0.057
High school diploma or equivalent	8.12	2.24	<0.001
Bachelor’s degree	4.65	1.95	0.017
Major (*ref. Health-related*)			
Non-health related	3.79	1.44	0.008
Governorate (*ref. Mount Lebanon*)			
Beirut	2.31	1.67	0.167
Bekaa/Baalbeck hermel	5.49	1.93	0.004
South Lebanon Nabatieh	5.73	1.95	0.003
North Akkar	2.76	3.46	0.425

These findings underscore the role of education in promoting informed perspectives on GMFs and highlight the value of tailoring educational content to fill knowledge gaps across different demographics. Future educational interventions could further explore tailored approaches to address preexisting disparities, ensuring that knowledge enhancements are both widespread and enduring.

### Attitudes and Perceptions Toward GMFs

**Figure 2.**
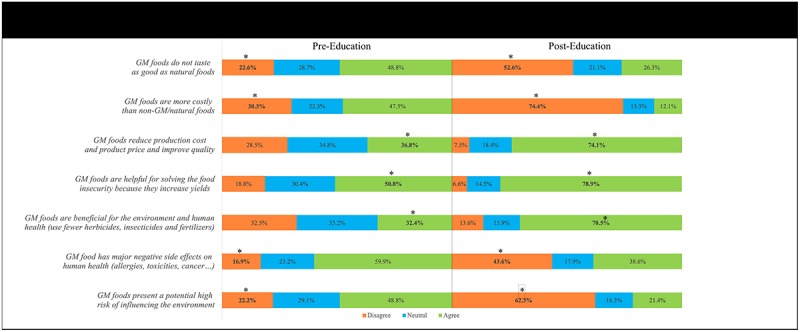
Comparison of pre-educational and post-educational attitudes and perceptions regarding GMFs.

Seven questions were designed to evaluate participants’ attitudes and perceptions regarding GMFs, with pre- and post-educational responses summarized in Figure 2. Overall, our participants displayed positive attitudes and perceptions toward GMFs, aligning with findings in the literature by Ghoochani et al. (2018)^34^, Ghanian et al. (2016)^35^, Prokop et al. (2013)^36^, Zammit-Mangion et al. (2012)^37^, and Heiman et al. (2011)^38^. Also, a meta-analysis of 25 studies on GMFs showed that consumers in various countries placed 42–23% higher value for GMF compared to non-GMF.^39^ In our study, 50.8% of participants agreed that GM technology increases yield, consistent with Catherine et al.^3^ who reported that 48% of study participants in Kenya viewed GM technology as important for boosting agricultural productivity. In contrast, 32.4% of our sample believed that GMFs require fewer herbicides, insecticides, and fertilizers, compared to 68.5% in Kenya. Moreover, 48.8% felt that GMFs do not taste as good as natural foods, while 50% of Kenyan participants reported enhanced taste, flavor, and appearance in GMFs. Furthermore, 36.8% of our participants believed that GM technology improves quality, compared to 47.7% in the Kenyan study, and 36.8% viewed GMFs as more affordable, versus 43.1% in Kenya. Lastly, 59.9% of our participants agreed that GMFs have major negative side effects on humans, whereas, in Israel, GMFs were perceived to be healthier than their conventional counterparts by the majority of participants.^40^ Remarkably, the percentage of participants who showed a positive attitude or perception toward GMFs increased across all questions following the educational component.

*Correct attitude

The mean attitude/perception scores improved significantly (*p* < 0.001), increasing from 30.3% ± 25.1 before to 38.9% ± 12.4 after the educational session. Indeed, before the educational intervention, higher educational levels were significantly associated with higher mean attitude/perception scores (*p* < 0.001), while unemployment correlated with lower scores (*p* = 0.027). On the other hand, participants with a health-related educational background scored significantly higher (*p* < 0.001) only on the pre-educational assessment. Interestingly, the mean attitude/perception scores increased following the educational component irrespective of the independent variables (Table 6).

**Table 6.** Relations between demographic characteristics and mean GMF attitudes/perceptions score before and after the educational session.

Demographic Characteristics	Before		After	
	Mean	SD	Mean	SD
**Gender**				
Male	28.9	24.6	38.9	13.2
Female	31.0	25.3	38.9	11.9
*Sig.*	0.22		0.97	
**Residential Governorate**				
Beirut & suburbs	32.0	24.1	40.2	13.1
Mount Lebanon	32.3	26.1	37.4	12.9
Bekaa/Baalbeck-Hermel	28.3	25.6	40.6	10.3
South Lebanon/Nabatieh	26.9	25.1	37.8	11.9
North Lebanon/Akkar	28.6	22.0	33.7	12.5
*Sig.*	0.088		<0.001	
**Educational Level**				
Uneducated	14.9	24.0	33.9	16.2
Elementary/Primary school or equivalent	20.9	25.0	39.2	12.9
High school diploma or equivalent	26.3	22.9	38.5	11.5
Bachelor’s degree	32.2	25.7	39.2	12.5
Master’s degree	33.9	23.4	38.4	11.6
Doctorate degree	35.9	25.8	41.7	13.2
*Sig.*	<0.001		0.262	
**Age**				
18–24	32.1	25.9	39.3	12.1
25–34	29.0	24.1	39.0	13.0
35–44	25.4	24.6	39.9	14.4
45–54	31.4	23.8	36.5	10.7
55–64	25.0	24.0	39.5	12.3
65 and above	27.3	20.6	37.3	13.6
*Sig.*	0.052		0.245	
**Major of study**				
Health-related	32.1	25.9	38.8	10.8
Non-health related	29.0	24.1	39.4	11.9
*Sig.*	<0.001		0.571	
**Area of residence**				
City	30.5	24.7	39.1	12.4
Town	32.0	26.2	39.6	10.4
Village	26.5	25.1	37.0	14.6
*Sig.*	0.151		0.166	
**Working status**				
Unemployed/Retired	26.4	22.6	37.3	11.9
Student	31.9	26.3	39.3	12.1
Employed/Self-employed	30.2	24.3	39.3	13.1
*Sig.*	0.027		0.136	
**Field of work**				
Health-related	34.1	26.5	40.1	13.0
Non-health related	31.6	23.0	39.6	12.5
*Sig.*	0.58		0.848	

The significant improvement in mean attitude/perception GMF scores from 30.3% to 38.9% following the educational component highlights the effectiveness of targeted education in shaping more informed and positive attitudes toward GMFs. This aligns with existing literature, which demonstrates that educational interventions can reduce misunderstandings and improve acceptance of scientific topics like GMOs.^38^ Notably, before the educational intervention, higher educational levels were associated with more favorable attitudes, suggesting that individuals with more advanced education may be better equipped to interpret scientific information, including the benefits and risks associated with GMOs. In agreement with these findings, our data showed a positive moderate correlation (*r* = 0.347; *p* < 0.001) between knowledge scores before education and mean attitudes/perceptions scores. These results further support the positive influence of knowledge and educational level on the individuals’ attitudes/perceptions toward GMFs, but also highlight the contribution of other factors, such as culture, beliefs, and government trust, to developing a positive attitude/perception and a better acceptance rate to consume GMFs.^13,19,41^ Conversely, unemployed participants scored lower on attitudes and perceptions, which could indicate limited exposure to structured learning environments where such topics are discussed, underscoring the importance of accessible educational outreach. It is worth highlighting that 25.3% and 52.1% of the participants stated their willingness to eat GMFs before and after the educational intervention, respectively. This shows the importance of education in changing the perceptions of the public to GMFs, and subsequently their acceptability toward GMF purchasing and consumption.

Strengths of our study include a comprehensive 50-item questionnaire adapted to the Lebanese context, ensuring relevance and accuracy, and bilingually translated and pilot tested, enhancing the clarity and validity of responses. Additionally, while a notable portion of participants who were initially approached dropped out of the study, our sample of 1,001 participants from various Lebanese governorates is still considered representative, increasing the generalizability of the findings across the Lebanese population. Furthermore, the inclusion of a structured educational session allowed the study to assess the impact of education on participants’ knowledge, attitudes, and perceptions of GMF, providing valuable insights into the effectiveness of information dissemination. By evaluating participants’ responses before and after the educational component, the study effectively measured changes in understanding and attitudes, highlighting areas where knowledge gaps were bridged. Finally, this study addresses a gap in GMF research within the Arab region, specifically Lebanon, where such information is limited. It would be interesting to explore in the future the sources of information on GMFs and the factors that influence individuals’ perceptions and attitudes, such as media exposure, ethical concerns, and cultural beliefs, as these could provide additional context for our findings.

## 4. Conclusion

This study, the first-of-its-kind in Lebanon, assesses knowledge, attitudes, and perceptions regarding GMFs, shedding light on critical socio-demographic disparities. Our findings demonstrate that tailored educational interventions can significantly improve knowledge and positively influence attitudes toward GMFs, particularly among groups with initially lower knowledge level, such as older individuals, those without graduate studies, and people with non-health-related educational backgrounds. These results emphasize the importance of targeted educational initiatives to bridge knowledge gaps and address misconceptions about GMFs. The implications of this study extend beyond Lebanon, providing valuable insights for other countries, particularly those in similar socio-economic and cultural contexts. Nations with diverse demographic profiles or limited public understanding of GMFs can adopt similar educational strategies to enhance awareness and promote evidence-based perspectives. By addressing socio-demographic factors that influence knowledge and attitudes, policymakers and educators can design more inclusive and effective programs tailored to their populations. Ultimately, this study underscores the transformative potential of education in fostering informed decision-making about GMFs, contributing to better public health policies and informed consumer choices. Further research is recommended to explore long-term retention of knowledge and to evaluate the effectiveness of educational interventions across different regions and cultural settings.
